# Comparative study of joint bioinformatics analysis of underlying potential of ‘neurimmiR’, miR-212-3P/miR-132-3P, being involved in epilepsy and its emerging role in human cancer

**DOI:** 10.18632/oncotarget.16541

**Published:** 2017-03-24

**Authors:** Lu Xia, Daojiang Li, Changwei Lin, Shuchun Ou, Xiaorong Li, Songqing Pan

**Affiliations:** ^1^ Department of Neurology, Renmin Hospital of Wuhan University, Wuhan 430060, Hubei Provinces, China; ^2^ Department of General Surgery, The Third XiangYa Hospital of Central South University, Changsha, Hunan 410013, China; ^3^ Center for Experimental Medicine, The Third XiangYa Hospital of Central South University, Changsha, Hunan 410013, China

**Keywords:** miR-132, miR-212, epilepsy, cancer, tumor-induced epileptogenesis

## Abstract

Considering the critical roles of miR-132/212 participated in central nervous system, many researches started to explored the contributions of miR-132/212 to epilepsy and achieve something worthwhile. Further illuminates all the genes targeted by miR-132/212 may be a valuable means for us to completely understand the working mechanism playing in epilepsy, by which it can influence diverse biological process. This study attempts to establish macrocontrol regulation system and knowledge that miR-212-3p/132-3p effected the epilepsy, for this literature search, miRbase, Vienna RNAfold webserver, Human miRNA tissue atlas, DIANA-TarBase, miRtarbase, STRING, TargetScanhuman, Cytoscape plugin ClueGO + Cluepedia+STRING, DAVID Bioinformatics Resources, Starbase, GeneCards suite and GEO database are comprehensive employed, miR-132-3p/212-3p and its target gene were found have highly expressed in brain and lots of molecular function and metabolic pathways associated with epilepsy may be intervened by it. Meanwhile, the emerging role of miR-132-3p/212-3p being involved in human cancer also been analyzed by several webtools for TCGA data integrative analysis, most remarkably and well worth exploring in our research conclusion that showed miR-132-3p/212-3p may be the core molecular underlying tumor-induced epileptogenesis.

## INTRODUCTION

Epilepsy is a common, serious neurological disorder characterized by recurring seizures due to abnormal neuronal excitability and which do latent harm to human health. However, the pathogenic mechanism resulted in epilepsy and resistance to currently available antiepileptic drugs (AEDs) remains poorly understood. Recently, accumulating evidence has found microRNAs (miRNAs) play pivotal modulators in pathogenesis and potential treatment for epilepsy [1]. In pathogenesis, miRNAs is thought to associate with large-scale changes to expression of gene modulating neuronal microstructure, cell death, neurotransmitter signaling, ion channels, gliosis, inflammation [2, 3], which indicating potential for miRNA-based therapeutics in epilepsy. Furthermore, Biofluids miRNAs profiles can also be utilized as useful biomarkers of epileptogenesis, disease risk and treatment assessment [3, 4]. miR-212/132 gene cluster is one of typical example.

MiR-132 and miR-212 were first discovered in mice and display brain and testes tissue-specific patterns of expression [5]. Has-miR-132 and has-212, similarly to their rodent orthologues, share the same primary transcript and first identified in neuronal cells from a screen designed to discern genes mediated by the cAMP-response element binding (CREB) protein transcription factor which is important for neuronal development and function [6]. Then, a great deal of studies have demonstrated that marked increase in transcription from the miR-132/212 locus can be caused by neuronal stimulation, and the expression of miR-132 and miR-212 is necessary for the proper development, maturation, morphogenesis and function of neurons and whose dysregulation has more to do with a large amount of neurodegenerative disorders, such as tauopathies, schizophrenia, Alzheimer's disease, Huntington's disease, autism and the theme of this article, epilepsy. (Discussed in two excellent reviews [7, 8]). Given this, miR-132/212 sometimes classified as ‘neurimmiR’ [8]. Although their role in neuronal functions is the most studied, more evidences point towards an involvement of these miRNAs in human cancer have been found such as miR-212 may improve the current prognostic risk stratification of mixed acute myeloid leukemia [9], epigenetic regulation of miR-212 expression in lung cancer [10] and down-regulation of microRNA-132 is associated with poor prognosis of colorectal cancer [11].

In most cases, miRNAs performed its regulating function in virtue of its target gene by a complementary manner, in which miRNAs guide RNA-induced silencing complex (RISC) to miRNAs response elements on target transcripts and usually lead to degradation or translational inhibition of their mRNA targets [12]. In humans, the miR-212/132 gene cluster exhibit similar mature sequences and share identical 8 base identical seed sequences, by which they may therefore target the same mRNAs and then involved in translational inhibition of these target genes [7]. In this maner, both miRs can regulate plenty of distinct gene target, and aid in the coordinate regulation of members of physiological and pathological process [13]. So, studying all genes directly target by miR-212/132 is important for us to understand thoroughly how they are involve in pathological mechanism of neurological disorders like epilepsy.

In the post-genomic era, a large amount of long-accumulated genomic data and data mining techniques for the life sciences provide us means to systematically understand the complex gene regulatory networks [14]. So, bioinformatics analyses were used in this study to better understand miR-212/132 gene cluster and its gene regulatory network, and combing the literature review and clinical database to explore the novel and worthwhile studying direction for epilepsy research. In the process of analysis, the regulatory network involved in other disease will often inadvertently be excavated out.

## RESULTS

### Mature sequence hsa-miR-132-3p and has-miR-212-3p may have a predominant role in nervous tissues

**Figure 1:**
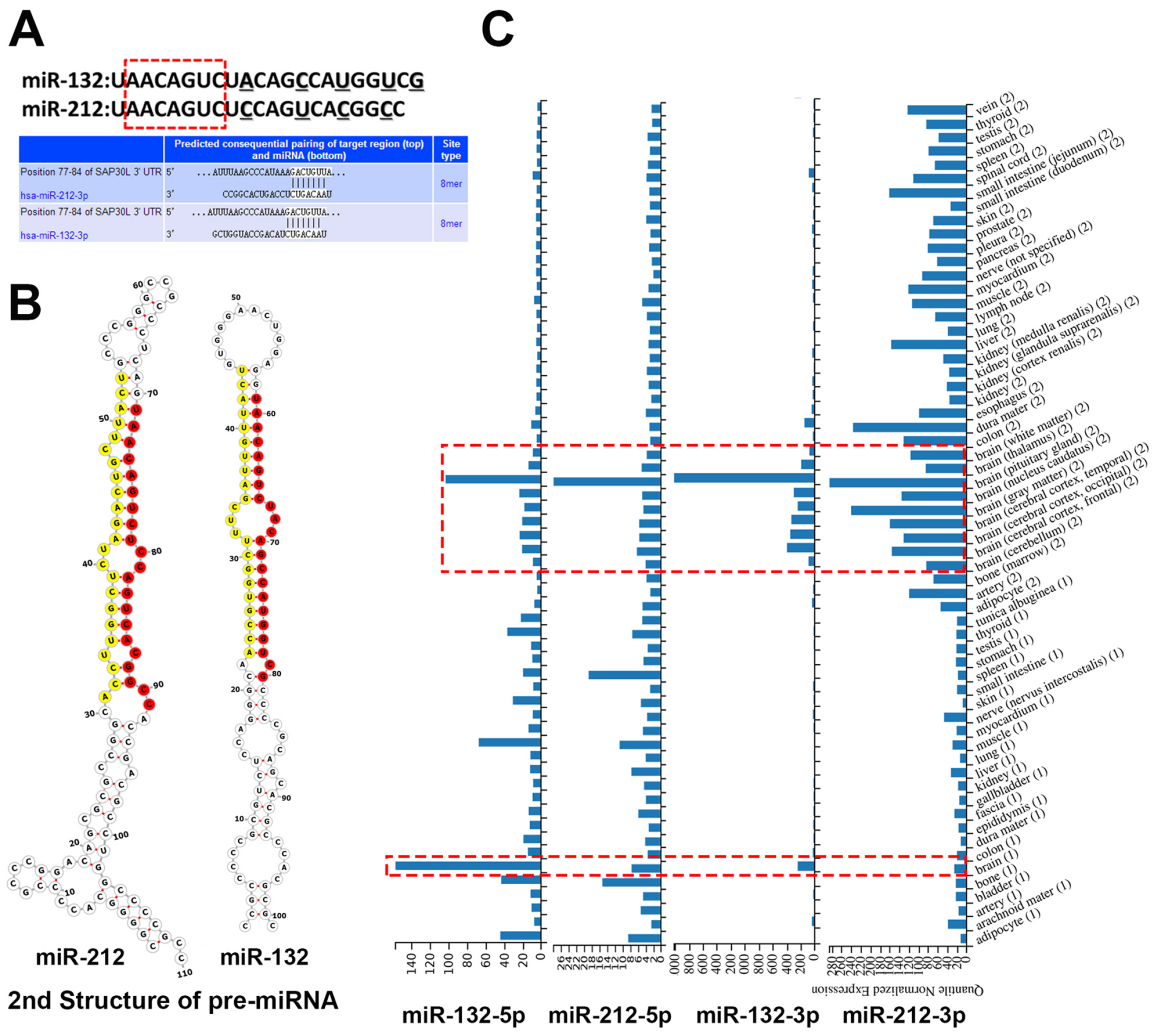
The gene sequence of miR-132/212 and its expression in human tissues **(A)** the similar mature sequences of miR-132 and miR-212, underline letters are the diverging nucleotides between the two human sequences, the sequence with red frames are common base seed sequences of both miRs. The genes SAP30L as an example illustrate base-complementation of both miRs. **(B)** the Sequences and predicted 2nd structure of human pre-miR-132 and pre-miR-212, the mature miR-132-3p and miR-212-3p are indicated in red, and the-5p are indicated in yellow, miRBase and Vienna RNAfold webserver are employed for these result. **(C)** the Human miRNA tissue atlas demonstrated that miR-132-3p/5p and miR-212-3p/5p have a high concordance of brain tissue-specific expression in human (red dashed box).

“miR-132” and “miR-212” are the most frequently used name in the vast majority of researches, but two mature microRNAs (miR-132-3p/5p and miR-212-3p/5p, respectively) originate from opposite arms of the same pre-miRNA have been found in human genome, so first we need to clarify which one is usually much more abundant in cell and on behalf of the “miR-132/212”, eventually, although there is also a small study miR-132-5p/212-5p oriented, miR-132-3p and miR-212-3p was determined as the predominant by miRbase and sequences alignments in published literature. Furthermore, miR-132-3p and miR-212-3p share similar mature sequences and common target gene, which is important for further analysis. Figure 1A/1B presented the mature and precursor sequences of miR-132 and miR-212 in human and one example about common target gene of both miRs. Knowing the expression and distribution of miRs in different tissues is essential for understanding normal and disease development of respective tissue. Such as miR-1/133a and miR-206/133b, which are highly expressed in myocardia and muscle, and are well characterized as muscle-specific miRNAs (myomiRs) that regulate key genes in muscle development [15]. So we want to make clear whether the expression of miR-132-3p and miR-212-3p have got relatively nerve tissue specifity, the data coming from the Human miRNA tissue atlas showed that miR-132-3p and miR-212-3p have a high concordance of brain tissue-specific expression in human, which indicated these miRs play a pivotal role in brain tissue development and impaired expression of them may result in disorders of the nervous system. (Figure 1C)

### Experimentally validated target genes of miR-212-3p/miR-132-3p have highly expression in brain

Before comprehensive target gene analysis, it's important to define the present research situation of experimentally validated miRNA:gene interactions. Table 1 and Supplementary Table 1 presented experimentally validated target gene of miR-212-3p/miR-132-3p, there are 951 genes have been validated and all of them have been discussed in various human diseases. Because miRs perform functions mainly depending on the sequence complementarity of seed regions [16], so all these validated miRNA:gene interactions theoretically has the possibilities correlated with the pathogenesis of many neural diseases. The fact showed in Table 2 and Supplementary Table 2 that most of these genes were highly expressed in brain make the role of miR-212-3p/miR-132-3p as “neurimmiRs” becomes even more prominent.

**Table 1:** Analysis of experimentally validated target gene of miR-212-3P/miR-132-3P

Database	miRs	Sum	Repetition	Remaining	combination 1	combination 2
**Tarbase**	miR-132-3p	723*	2	721	898	951
	miR-212-3p	560*	0	560		
**miRTARBASE**	miR-132-3p	263	28	235	248	
	miR-212-3p	99	22	77		
**miRecored**	miR-132-3p	1	0	1	2	
	miR-212-3p	1	0	1		
**miRpathDB**	miR-132-3p	235	0	235	248	
	miR-212-3p	77	0	77		

723* include 88 genes genes of validation type being indirect or unkonwn;560*:include 2 genes of validation type being indirect or unkonwn; combination 1:Combinate the target genes of both miRs and remove duplicates; Combination 2: Combinate all target genes of all database and remove duplicates

**Table 2:** the experimental validated target genes have highly expressed in brain (DAVID was used and the top 10 is listed in this table)

Category	Term	Count	%	PValue	Benjamini	FDR
UP_TISSUE	Epithelium	245	26.18	6.62E-22	1.83E-19	8.73E-19
**UP_TISSUE**	**Brain**	**489**	**52.24**	**5.7E-10**	**7.94E-08**	**0.000000757**
UP_TISSUE	Uterus	141	15.06	2.01E-08	1.85E-06	0.0000265
UP_TISSUE	Ovarian carcinoma	26	2.78	5.71E-07	0.0000395	0.000754
UP_TISSUE	Platelet	54	5.77	5.89E-06	0.000326	0.007770269
UP_TISSUE	Placenta	219	23.40	5.22E-05	0.0024082	0.068915566
UP_TISSUE	Human uterus endothel primary cell culture	6	0.64	5.31E-05	0.0020975	0.0700183
UP_TISSUE	Cajal-Retzius cell	27	2.88	5.75E-05	0.0019892	0.075882518
UP_TISSUE	Uterus endothel	8	0.85	7.36E-05	0.0022629	0.097117051

### Computational analysis result of miR-132-3p and miR-212-3p-regulated biomolecular network

**Figure 2:**
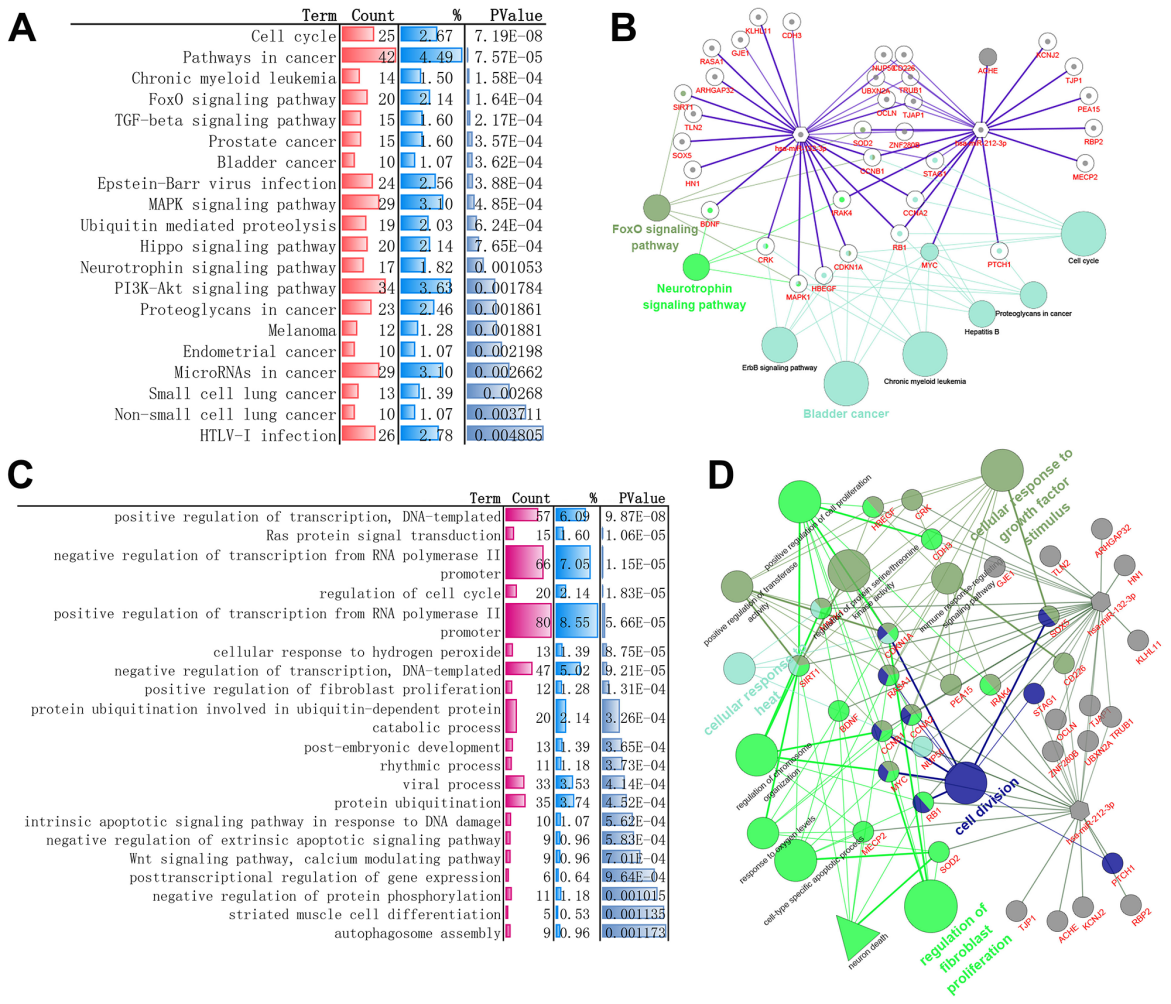
Experimental validated Gene-enrichment analysis result of miR-132-3p and miR-212-3p-regulated biomolecular network **(A)** Top 20 statistically enriched KEGG pathway (DAVID, more details can be found in Supplementary Table 3). **(B)** Representative cases about 20 validated genes (in red) targeted by both miRs and corresponding pathway analysis against KEGG database. (miRtarbase, KEGG database). **(C)** Top 20 statistically enriched biological processes categorization (DAVID, more details can be found in Supplementary Table 3). **(D)** Representative biomolecular network about 20 validated genes (in red) targeted by both miRs and corresponding biological process against GO Consortium. (miRtarbase, GO).

The occurrence of any disease involved some physiological and biochemical processes, such as biological process, molecular function, cellular component and metabolic pathways, these biological mechanism enrichment can provide clues and references for further research, so Gene Ontology classification(biological process) and KEGG Pathway enrichment analysis of these validated genes was performed. The result of KEGG pathway revealed that these genes might be associated with 41 statistically enriched categories, such as cell cycle, foxO signaling pathway, TGF-beta signaling pathway, MAPK signaling pathway, neurotrophin signaling pathway and several pathway about cancers. Biological process such as transcription, cell cycle, positive regulation of protein export from nucleus, synapse assembly, axon extension, brain development, axon regeneration are statistically enriched. Among these, transcriptions are primary regulation process such as “positive regulation of transcription, DNA-templated”, “negative regulation of transcription from RNA polymerase II promoter” and “positive regulation of transcription from RNA polymerase II promoter”. more details can be found in Figure 2 and Supplementary Table 3)

### The biomolecular information of remaining genes targeted by miR-132-3p and miR-212-3p

**Figure 3:**
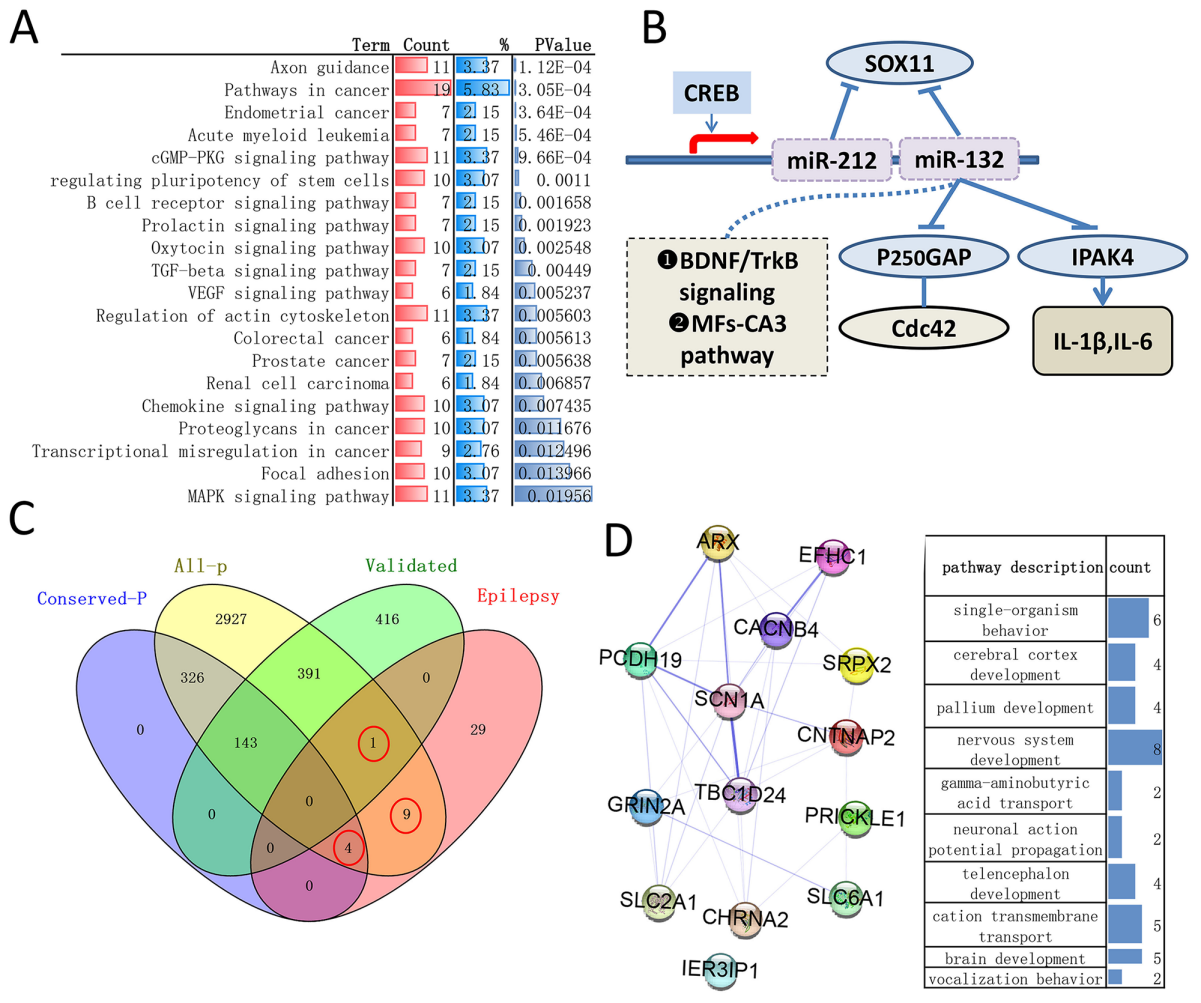
The biomolecular information of remaining genes and the role of miR-132, miR-212 and genes participate in epilepsy **(A)** Top 20 statistically enriched KEGG pathway (DAVID, more details can be found in Supplementary Table 4). **(B)** The mechanism that miR-132/212 was involved in epilepsy are briefly summarized in this diagram (literature search). **(C)** Venn diagrams of calculating the intersection (s) of four groups of genomes (analyzed by Venny 2.1), all-P: all predicted targets of Targetscan 7.1, irrespective of site conservation, conserved-P: all predicted targets with conserved sites by Targetscan 7.1, validated: experimental validated genes, epilepsy: the genes related to epilepsy sourced directly from MalaCards human disease database. The red cycle indicated the intersection genes. 9 in all-P, 1 in validated and 4 in conserved-P. **(D)** Functional enrichments of 14 intersection genes by web-STRING and Cytoscape plugin STRINGApp, biological process (right) and protein-protein interactions network (left). More details about analysis result can be found in Supplementary Table 5.

Meanwhile, all predicated targets but not been experimentally validated genes may have potential research values in recent years, so Targetscan, a target predicting programs with higher sensitivity and precision than other [17], was employed to analyze all predicted gene. Here, we focus on the transcripts with conserved sites, as shown in Figure 3C and Supplementary Table 4, 473 genes (474 genes identified firstly but with one repeat) were identified and remaining gene is 330. The statistically enriched pathway of these remaining genes showed axon guidance and pathways in cancer ranked first two, biological process categorization showed that significant portions of the genes were grouped as transcription, biosynthetic process, metabolic process and regulation of gene expression (Figure 3A and Supplementary Table 4). It should be noted that all analysis of biological networks for target genes have identical result in ClueGO + Cluepedia, DAVID and STRING (data not shown).

### The emerging role of miR-132 and miR-212 participate in epilepsy

All target genes and whose biological process had been enumerate above, in order to better understand the mechanism specific to epilepsy, literature searches (pubmed) was employed and the mechanism that miR-132/212 was involved in epilepsy are briefly summarized in here. MiR-132/212 both were highly expressed in experimental and human epilepsy [3], CREB-regulated microRNA miR-132 can be rapidly induced by activation of neurons in vivo [18] and the p-CREB and miR-132 were highly expressed in both rats and patients with temporal lobe epilepsy (TLE)[19]. MiR-132 is important regulators of seizure-induced neuronal death [20] and whose silencing inhibit the spontaneous seizures through the MFs-CA3 pathway [21]. miR-212-3p and miR-132-3p work synergistically to control Sox11 expression in the setting of epilepsy [22]. miR-132 play a negative feedback regulator of IL-1β and IL-6 by targeting IRAK4 in astrocyte-related inflammation induced by MRP8 [23] and promotes epileptogenesis by modulating BDNF/TrkB and p250GAP/Cdc42 signaling in the hippocampal neuronal culture model [24, 25].Figure 3B

### The genes may be related to biomolecular pathways of epilepsy

The MalaCards human disease database integrates both specialized and general disease lists and can provide the list of affiliated genes, pathways, gene ontologies (cellular component, biological process and molecular function) associated with the key disease [26]. “Epilepsy” is used for search, 43 genes (such as the gene, EPM2A, presented a relevance score of 88.82) compiled from “Gene cards” and “Disease” database are related to epilepsy (Supplementary Table 5). Benzodiazepine Pathway, Pharmacodynamics and locomotion are the top 1 pathways and GO terms (Biological processes) related to epilepsy, respectively (more details can be found in MalaCards). In this article, we want to assure whether these genes significantly associated with epilepsy can be directly and indirectly affected by miR-132-3p and miR-212-3p. Figure 3C demonstrated that 14 (32.6%, 14/43) genes may be targeted by both miRs. Among these genes, only *IER3IP1* have been validated, *SLC6A1, SCN1A, SLC2A1, ARX* have conserved sites,*CACNB4,CHRNA2 CNTNAP2, EFHC1, GRIN2A, PCDH19, PRICKLE1,SRPX2,TBC1D24* have poorly conserved sites. For conforming the fact that these genes did were implicated in epilepsy, STRING analysis was carried out and showed that the GO terms of 14 genes are markedly related to nervous system (Figure 3D, Supplementary Table 5). In addition, although 29 other genes related to epilepsy is the directly target genes in theory, we found several of them can be indirectly mediated by both miRs through protein-protein interactions and common biomolecular pathway (data not shown).

### miR-132-3p and miR-212-3p may play a critical role in human cancer

**Figure 4:**
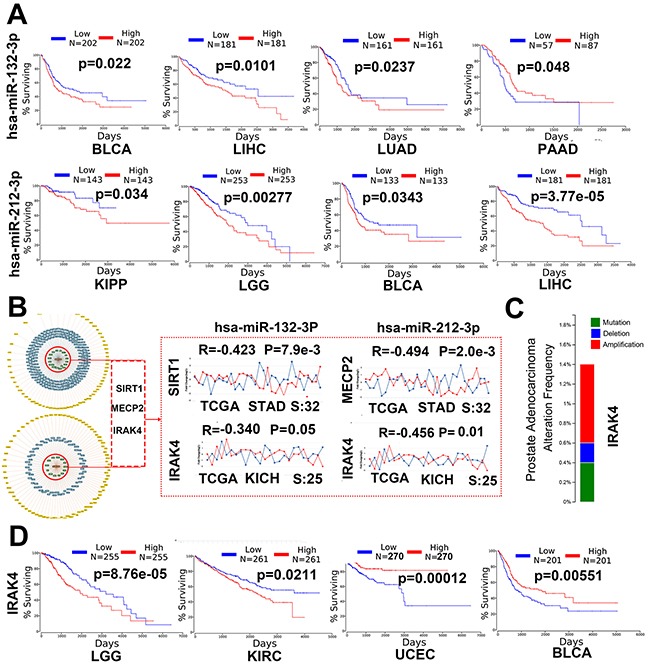
miR-132-3p and miR-212-3p may play a critical role in human cancer **(A)** Survival analyses of both miRs in different cancers. BLCA: bladder urothelial Carcinoma, LIHC: liver hepatocellular carcinoma, LUAD: lung adenocarcinoma, PAAD: pancreatic adenocarcinoma KIPP: kidney renal papillary cell carcinoma, LGG: brain lower grade glioma. **(B)** SIRT1, MECP2 and IRAK4 were selected as experimentally validated targets with strong evidence, Co-expression analysis of these genes and both miRs in stomach adenocarcinoma (STAD) and Kidney Chromophobe (KICH). **(C)** Somatic mutations analysis of IRAK4 gene through Cosmic (data not shown) and cBioportal in prostate cancer. **(D)** Survival analyses of IRAK4 mRNA expression in different cancers. LGG: brain lower grade glioma, KIRC: kidney renal clear cell carcinoma. UCEC: Uterine Corpus Endometrial Carcinoma, BLCA: Bladder Urothelial Carcinoma.

Gene ontology and pathway enrichment analysis about validated and remaining target genes indicated miR-132-3p and miR-212-3p may play a critical role in human cancer. For experimental validated genes and predicted genes with conservative sites, 34.9 %(15/43) and 38.2%(13/34) statistically enriched pathways are directly correlated with cancer, respectively, including Chronic myeloid leukemia, prostate cancer, bladder cancer, proteoglycans in cancer, Glioma, endometrial cancer, colorectal cancer and so forth. Literature search (pubmed) show that 26.1 % (95/364) (“miR-132”/“miR-132” and“cancer”) and 37.2 %(45/145) (“miR-212”/“miR-212” and“cancer”) studies are related to cancer for miR-132 and miR-212, respectively. Given all that, survival analysis sourced from TCGA data was performed and the result showed that miR-132-3p and miR-212-3p were presented to be up-regulated or down-regulated in different cancer types, and Kaplan-Meier analysis curves demonstrated that aberrantly expression of both miRs was conspicuously associated with poor overall survival (Figure 4A). Previous research have found that miR-212/132 have bilateral function depending on cancer types [7], which is in line with the our analytic result, such as high miR-132-3p and low miR-132-3p expression are significantly associated with poor survival in bladder urothelial carcinoma (BLCA) and pancreatic adenocarcinoma (PAAD), respectively. (Figure 4A).

### The core target genes of miR-132/212 were involved in human cancer

It is clear now that miR-132-3p/212-3p and whose biomolecular networks are markedly implicated in human cancer. To further understand the specific mechanism how both miRs are involved in cancer by suppress single target gene, 3 experimentally validated targets with strong evidence, i.e., SIRT1, MECP2,IRAK4,were chosen for co-expression analysis. Previous study coming from our and others have found miR-132 presented tumor-promoting but miR-212 served as tumor-suppressing feature in stomach adenocarcinoma (STAD) [27, 28], in here, the genes, i.e., SIRT1 and MECP2 with such similar reverse feature were shown to be down-regulated and up-regulated in STAD [29, 30], have conspicuously negative connection with miR-132-3p and miR-212-3p in STAD, which partially confirms the fact that both miRs have reverse functions in some tumors. Further studies are needed to do due to the same seed sequence of both miRS. Based on the fact that a large amount of biomolecular networks are associated with caner, we wonder whether there are common gene or pathway related to both miRs cause the epilepsy and cancer.IRAK4 clearly relevant to epilepsy assigned to further study, Figure 4B and Figure 4D show IRAK4 is negatively with the miR-132/212 in kidney chromophobe and aberrantly expression of IRAK4 are significantly related to poor survival of several cancers, certainly, somatic mutations of IRAK4 itself in cancer is another main reason in cancer (Figure 4C), but on the whole all of which suggest that miR-132/212 may be involved differently disease though single gene.

### miR-132/212 may be the core molecular underlying tumor-induced epileptogenesis

**Figure 5:**
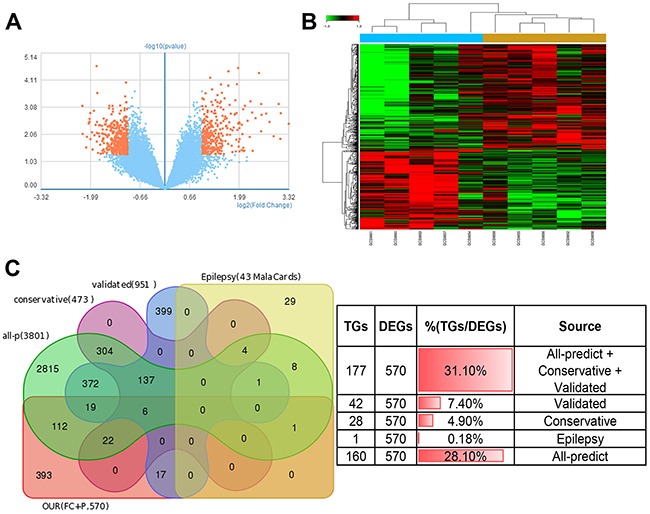
miR-132/212 may be the core molecular underlying tumor-induced epileptogenesis **(A)** the Valcano figure compiled from data of GSE32534, which demonstrated 744 probe sets representing 568 differentially expressed genes (DEGs) between the two groups of 5-seizure vs. 5-non-seizure in low grade brain tumor patients, the red indicate the DEGs(2-fold plus p≤0.05, no FDR applied). **(B)** the heatmap shows differentially expressed 744 probe sets compiled from data of GSE32534 (2-fold plus p≤0.05, no FDR applied). **(C)** Venn diagrams show the intersections of 5 groups of genomes, all-P: all predicted targets of Targetscan 7.1, irrespective of site conservation, conservative: all predicted targets with conserved sites by Targetscan 7.1, validated: experimental validated genes, epilepsy: the genes related to epilepsy sourced directly from MalaCards human disease database. OUR (FC+P): the DEGs between both group of 5-seizure vs. 5-non-seizure in low grade brain tumor patients, 568 genes coming from our analysis, 2 genes significantly related epilepsy coming from original article was supplemented (2-fold plus p≤0.05, no FDR applied). The table (right) indicated the number and portion of intersection genes.(TGs: target genes)

All the analyze result above, especially the fact that IRAK4 targeted by miR-132/212 can participate in cancer and epilepsy and the survival of brain lower grade glioma (LGG) is significantly affected by both miRs (P=0.002) and its target genes like IRAK4 (p=8.76e-05) (Figure 4B and Figure 4D) remind us whether miR-132-3p and miR-212-3p is the core molecular underlying brain tumor-induced epileptogenesis, To illustrate this hypothesis, GEO (access #: GSE32534), which was the first study of to use formalin-fixed paraffin embedded peritumoral tissues (5-seizure vs. 5-non-seizure)to investigate the global gene expression in low grade brain tumor patients with epilepsy, was download for further study. According to the description in original article [31], parametric no paired Student T-test (2-fold plus p≤0.05, no FDR applied) was used. 744 probe sets (representing 568* differentially expressed genes (DEGs), *the same gene with different probe sets was merged) were identified between the two groups, despite there's some discrepancy between our result and original paper (345 probe sets representing 296 genes), all 568 genes was believed for further analyze given that two result almost perfect overlap. (Figure 5A, 5B and Supplementary Table 6). Firstly, we want to know whether the 570(2 gens in Original article significantly associated epilepsy were include) DEGs can directly target by miR-132-3p and miR-212-3p, the intersections between DEGs and target genes derived from different source were calculated, Surprisingly and fascinating, venn diagram shown that 31.1%(177/570) DEGs may be target by both miRs and 7.4%(42/570) DEGs have been experimentally validated. (Figure 5C and Supplementary Table 6)

### The molecular mechanism underlying miR-132/212 implicated in tumor-induced epileptogenesis

**Figure 6:**
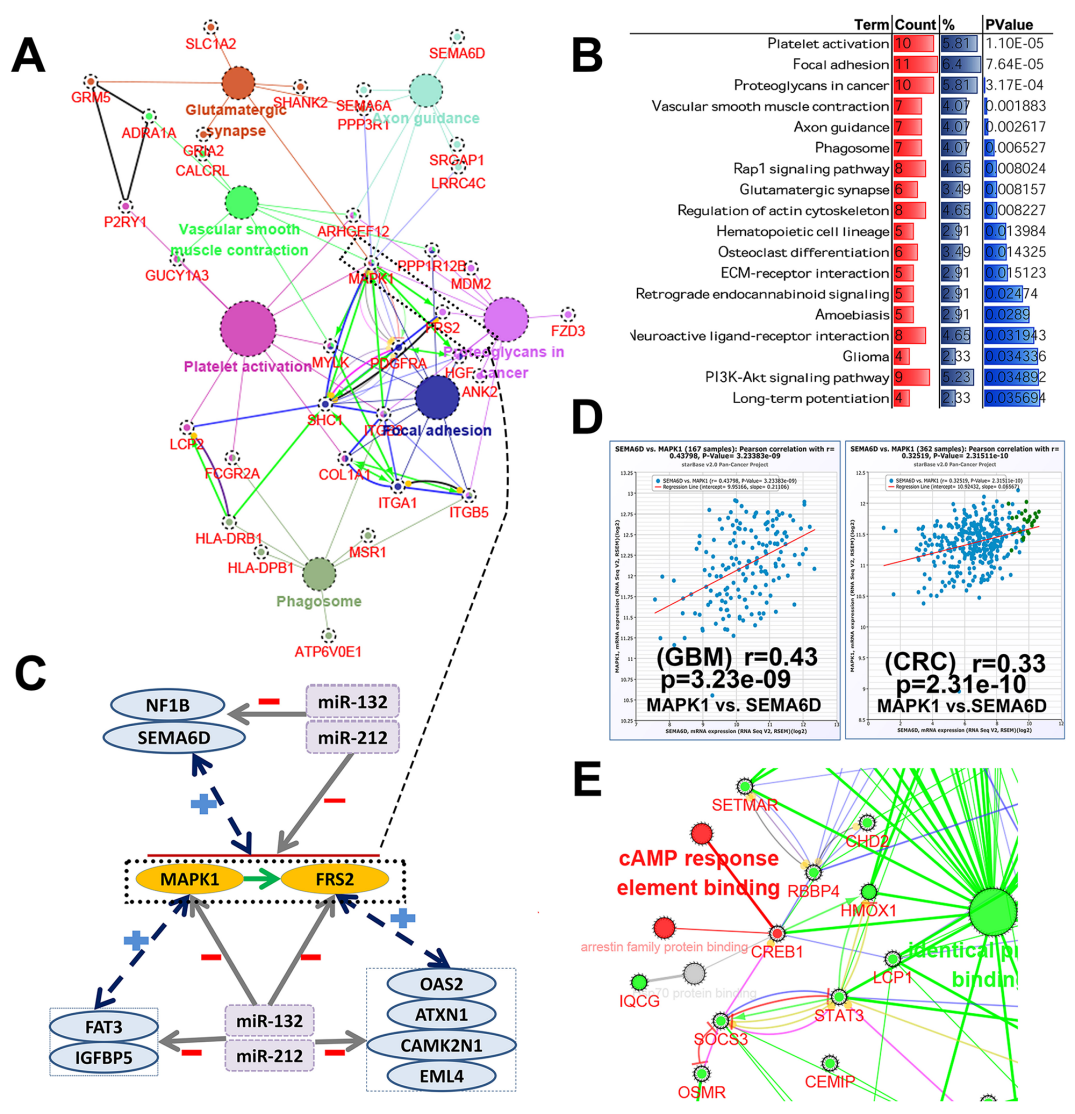
The molecular mechanism underlying miR-132/212 implicated in tumor-induced epileptogenesis **(A)** the biological network of 177 DEGs may be directly target by miR-132/212, the network consists of top 7 statistically enriched KEGG pathways (big cycle with different color) and corresponding genes (red) and whose STRING protein interaction (between the gene, the specific interactive mode will not discussed in here). **(B)** Top 18 statistically enriched KEGG pathway (DAVID, more details about KEGG pathway, GO-term and the tissue expression of DEGs can be found in Supplementary Table 7). **(C)** The simple simulated diagram for ceRANs network of 177 DEGs. The core gene MAPK1 and FRS2 coming from the network presented in Figure 6A was chosen as example, NF1B and SEMA6D was the common ceRNAs for both genes, FAT3 and IGFBP5 for MAPK1, and 0AS2, ATXN2, CAMK2N1 and EML4 for FRS2. **(D)** the representative example about the co-expression to support ceRNA hypothesis, positive co-expression of MAPK1 and ceRNA SEMA6D was shown in Glioblastoma multiforme (GBM) and Colon and Rectal adenocarcinoma (CRC)(more details can be found Supplementary Table 8). **(E)** CREB1, the transcription factor of miR-132 and miR-212, as the example to illustrate the complex indirectly regulatory networks that both miRs are implicated in remaining 68.9 %(393/570) DEGs without base-complementation with miRs, red cycle with red label indicate the molecular function and CREB1, green cycle and red label present the molecular function and corresponding remaining DEGs. (The Figure 6A and Figure 6E is designed by open source software Cytoscape 3.4.0 and its plugin or app ClueGO+Cluepedia)

The fact that both miRs have a high concordance of brain tissue-specific expression and large number of DEGs between low grade brain tumor patients with and without seizure can be directly controlled by them let us speculate miR-132/212 may be the core molecular underlying tumor-induced epileptogenesis. Then we were curious about the molecular mechanism how both miRs induced epileptogenesis in tumor, according to the main mechanisms of miRNA action, it's conceivable that 3 main mechanisms, i.e., direct intervention, ceRNA network and indirect adjustment, may be involved it. (1) 31.1% of DEGs can be directly target by both miRs is the best evidence for direct intervention, as demonstrated in Figure 6A/6B and Supplementary Table 7, pathway analysis against KEGG showed these DEGs (177) were statistically enriched in platelet activation, focal adhesion, proteoglycans in cancer, axon guidance, phagosome, glutamatergic synapse, neuroactive ligand-receptor interaction, long-term potentiation and glioma, besides, the network (Figure 6A) consists of KEGG pathway and STRING protein interaction revealed that MAPK1, FRS2, FDGFRA, SHC1, ITGB2 and ITGA1 may be the central genes for these pathway which miR-132/212 was involved in tumor-induced epileptogenesis directly. In addition, more evidence derived from GO term and other feature for these DEGs can be found in Supplementary Table 7. (2) Because identical seed sequences, the relative redundancy of miR-132 and miR-212 functions is still an incompletely addressed question. But in recent years, with the a large number of studies focus on ceRNAs network and found the range and strength of ceRNA regulation are largely determined by the relative abundance of miRNA [32, 33], we bravely assumed that both miRs may supplemented each other to maintain the abundance in ceRNA network. To look for evidence, 31.1 %(177/570) DEGs may be target by both miRs was used for ceRNA nework analysis, as demonstrated in Figure 6A/6C/6D and Supplementary Table 8, the core genes MAPK1 and FRS2 were random select for this study, the result compiled from Starbase displayed that a rich ceRNAs network for both genes, but more notably is most of the ceRNAs isn't appeared in statistically enriched pathway, which indicated ceRNAs like SEMA6D and NF1B are also the regulatory network in tumor-induced epileptogenesis. (3) CREB1, as the transcription factor and first identified the miR-212/132 in neuronal cells [6], serve as an example to illustrate the complex indirectly regulatory networks that both miRs are implicated in remaining 68.9%(393/570) DEGs without base-complementation with miRs, as displayed in the Figure 6D, Go term (molecular function) and STRING protein interaction network found STAT3, SOCS3, RBBP4, SETMAR, LCP1 and HMOX1 may effected the CREB1 to some extent, and further mediated the transcription of miR-212/132. In addition, that co-expression analysis found some gene present positive relationship with both miRs (data not shown). Other evidence like KEGG and STRING network consist of all the possible transcription factor compiled from Genecards and DEGs showed in Supplementary Table 8.

## DISCUSSION

In this study, hsa-miR-132-3p and has-miR-212-3p considered as one entire piece and on behalf of miR-132/212, the primary reason is that miR-132 and miR-212 share similar mature sequences and identical 8 base seed sequences, which is the key factor in base-complementation mechanism. Certainly, two things are worth mentioning when considering further discussion, firstly, miR-132 and miR-212 also presented different expression and miR-132 may be play a major role, each of these miRNAs respectively repress specific targets(diverging nucleotides between miR-132 and miR-212 sequences (see Figure 1A) and have a nonsynchronous status [16, 34]. The second one is that miR-132-3p/5p and miR-212-3p/5p, originating from opposite arms of the same pre-miRNA and with different seed sequence, may be involve in another unique role in CNS (figure 1C have showed 5p also have a high concordance of brain tissue-specific expression in human) [35–37].

When miR-212-3P and miR-132-3P determined as discussion objects, we firstly analyze the expression of miRNA and its target genes across human tissues, the result provide a sound basis for its involvement in neurological diseases such as epilepsy that miR-132/212 presents brain tissue-specific and elevated expression, there are far more similar example like miR-1/133a and miR-206/133b cluster, known as myomiRs, that suppress key genes in muscle development [15]; the liver-specific miRNA-122* and miR-122 participates extensively in human hepatocellular carcinoma [38, 39].

Because miRNAs execute functions through target genes and corresponding biological processes that these target genes involved in [12, 40], so we next study the target genes and enrichment of gene-GO term and bio-pathways. For better defined, the experimentally validated genes were studied firstly. The result demonstrated several enriched pathways have been previously implicated in epileptic seizure or related activities, such as decreased interaction between *FoxO3a* and *Akt* correlates with seizure-induced neuronal death [41], and *neurotrophin signaling pathway* in aging, Alzheimer's disease (AD), and other disorders such as temporal lobe epilepsy [42]. In addition, we found that regulating transcription is the primary biological process of miR-212/132 in enrichment analysis of GO, which is similar to the fact that microRNAs may play important role in transcriptional regulation [43]. To achieve a more comprehensive analysis of target gene, the same analysis was carried on remaining predicted targets, *Axon guidance* ranked first in these genes, previous study have found hippocampal axon guidance can be regulated by nor-1 and involve in seizure susceptibility [44], and *mTOR signaling pathway* can sever as a new therapeutic strategy in epilepsy and epileptogenesis, all which absented in validated target genes may be provide a valuable reference and guide for future study on miR-212/132in epilepsy.

Since so many biological mechanism are related to epilepsy, we are interested in the current research status that miR-132/212 had been involved in epilepsy, Pubmed search found 15 papers about these neurimmiRs participated in epilepsy, such as BDNF/TrkB pathway and p250GAP/Cdc42 signaling have been researched, BDNF/Trkb can be adjusted by *PI3K* and *MAPK* pathways, which is accorded with the above analysis. Comprehensive comparison, we feel that the study on involvement of both miRs in epilepsy is still less, there is still a large research space. Certainly, epilepsy is also belong to CNS disorder and results from a variety of CNS insults, theoretically, all disease caused by dysregulated expression of miR-132/212 can become the trigger factors for epileptic seizures.

In addition to Pubmed search, the MalaCards disease database [26] with high credibility and having been used widely was also used for further analysis of research potential that miR-132/212 can cause epilepsy, the MalaCards provides 43 affiliated genes found to be associated with the epilepsy, what has specific value in this article is that 32.6% of genes related to epilepsy may be directly targeted by miR-132-3p and miR-212-3p. Meanwhile, the fact that only one gene had been experimentally validated remind us that much work need to do to make up some deficiencies in this field.

Although in the beginning we did not take cancer as the main research object, as data are accumulated and analysis continues, the result demonstrated miR-132-3p and miR-212-3p may participates extensively in human cancer, For experimental validated genes and predicted genes with conservative sites, 34.9 % (15/43) and 38.2% (13/34) pathway are statistically enriched in cancer directly, respectively. Such as pathways in cancer, MicroRNAs in cancer and Proteoglycans in cancer, which is consistent with previous literatures about the emerging role of miR-132/212 involved in cancer. Survival analysis reveals that dysregulation of miR-132-3p and miR-212-3p is conspicuously related with poor survival in several human cancer including BLCA, PAAD, KICH and LGG. Moreover, both miRs may perform tumour-promoting and tumour-suppressing dual functions depending on cancer types, which is consistent with what been previously reported [7]. Our group also found both miRs present different expression and mechanism in STAD [27]. All unveil the complexity of both miRs involvement in tumourigenesis.

Though the co-expression analysis of miR-132/212 with selected core validated target genes, the double role of miRs functioned in caner been further validated. What is important is IRAK4, a genes associated with epilepsy [23], presented correlation with both miRs and whose dysregulation significantly related to poor survival in several human cancer. Apart from the somatic mutations of IRAK4 itself, all indicated miRs may target single gene to interfere the process of cancer and epilepsy. Certainly, as epilepsy-associated genome is rapidly increase, the tally of genes related to seizures will likely match and overlap that of cancer and beyond it in biological diversity [45]. Meanwhile, the fact that the survival of brain tumors such as LGG can be determined by miR-132 and the validated target gene IRAK4 and the incidence of epilepsy is much higher in low-grade gliomas than in high-grade gliomas [31] remind us whether miR-132/212 get involved in brain tumor-induced epilepsy.

GSE32534 which originally designed to explore the change of gene expression in peritumoral cortex tissue slides from 5-seizure vs. 5-non-seizure low grade brain tumor patients [31] was used for validated this hypothesis- miR-132/212 get involved in brain tumor-induced epilepsy. Fascinating thing is 31.1% DEGs may be directly target by both miRs, and further mechanism study indicated miR-132-3p and miR-212-3p may be participated in brain tumor-induced epilepsy through direct intervention, ceRNA network and indirect adjustment. Platelet activation, focal adhesion, proteoglycans in cancer, axon guidance, phagosome, glutamatergic synapse and MAPK1, FRS2, FDGFRA, SHC1, ITGB2 and ITGA1 may be the core bio-pathway and genes involved in direct intervention, respectively. Some genes did not appear in statistically enriched pathway and GO-term can sever as ceRNAs to compete for miR-132/212 and indirectly inhibit core gene such as NF1B, FAT3 and EML4. In addition, indirect adjustment may also play a role; some DEGs, such SOCS3 and RBBP4, without base-complementation with miRs can affected the CREB1 and then regulated transcription of both miRs. We would like to acknowledge that the number (3801) of possible target genes occupied a high percentage in genome, by comparison (data no shown), the proportion of genes related to miR-212/132 and have function in epilepsy at the same time is relatively high. So, these conclusions suggest both miRs are the core molecular for epilepsy.

## MATERIALS AND METHODS

### Analysis of gene structure and tissue specificity and heterogeneity of miR-212/miR-132

Stem-loop sequence has-miR-132 /212 generated two mature sequences, respectively. Previous study have demonstrated that although either strand of the duplex (miRNA:miRNA* duplex) may potentially act as a functional miRNA, only one strand will play the dominant role, so firstly, the miRbase [46] and Vienna RNAfold webserver [47] were used to identified the gene sequence and predicted stem-loop structures. miRNA research revealed different sets of miRNAs expressed in different cell types and tissue and have its specificity and heterogeneity, so Human miRNA tissue atlas [39] was adopted to certify the organ specificity of miR-132 /212.

### Analysis of experimentally validated target gene of miR-212-3P/miR-132-3P

Validated miRNA:gene interactions was conjointly analyzed by DIANA-TarBase v7.0 [48], miRtarbase [49], miRecored [50] and miRpathDB [51], all of which provide experimentally validated target gene. In addition, literature search was performed to supply latest and missing gene which may be left out by web-software.

### Analysis of biological networks for validated target gene of miR-212-3P/miR-132-3P

All enrichment analysis of gene-GO term and bio-pathways were identified statistically with the Cytoscape plugin ClueGO + Cluepedia+STRING app and DAVID Bioinformatics Resources 6.8 [40, 52, 53], STRING v10 [54] was used to provide a critical assessment and integration of protein-protein interactions. All gene tissue expressions in human tissue were assessed by DAVID Bioinformatics Resources 6.8. Besides, oriented to this article focused on epilepsy, literature search (PubMed) was carried out to analyze the exiting role of miR-132 and miR-212 involving in epilepsy.

### Analysis of all the target gene of miR-212-3P/miR-132-3P

TargetScanhuman7.1 was used to predicts biological targets of miR-212-3P/miR-132-3P, all remaining gene (refer to the gene that have ruled out the experimentally validated target gene from all predicted target gene) performed enrichment analysis of gene-GO term, bio-pathways as described above.

### Analysis of genes and related bio-pathways may be related to epilepsy using GEO data and MalaCards

To screen the differential expression gene between patients with and without epilepsy, GEO super-series GSE32534 were used to evaluated, processed and normalized expression data were download from NCBI and the bio-pathways of these differential expression genes was analyzed as above, Volcano Plot and dendrogram of different genes was analyzed through Gene-Cloud of Biotechnology Information (https://www.gcbi.com.cn/gclib/html/index), Meanwhile, in order to include more genes associated with epilepsy supportedby literature, the data compiled from MalaCards was employed [26]. Venn Diagrams and Venny 2.1 are used for calculating the intersection(s) of genomes. Function enrichment analysis is performed as above.

### Analysis of the emerging role of miR-132-3p and miR-212-3p involved in human cancer

Several Web based tools for “The Cancer Genome Atlas” (TCGA) have been used to visualize, analyze and interpret all the data types whether miR-132-3p and miR-212-3p may be involve in human cancer. Survival analyses for mRNAs ad miRNAs was performed on OncoLnc (http://www.oncolnc.org), Cosmic and cBioportal were employed for Mutation analysis [55] [56], Correlation analysis and competitive endogenous RNA (ceRNA) network was analysis carried out by miRtarbase, Starbase and cBioportal [57]. MiRTargetLink is used to select the experimentally validated targets with strong evidence [58].

## SUPPLEMENTARY MATERIALS TABLES


















