# Supplementary material for: Comparative study of joint bioinformatics analysis of underlying potential of ‘neurimmiR’, miR-212-3P/miR-132-3P, being involved in epilepsy and its emerging role in human cancer
Source: Oncotarget. 2017 Mar 24;8(25):40668–82. doi: 10.18632/oncotarget.16541 (PMC5522300; doi:10.18632/oncotarget.16541)
Supplement: Supplementary file 1 [file oncotarget-08-40668-s001.pdf]

Comparative study of joint bioinformatics analysis of underlying potential of 'neurimmiR', miR-212-3P/miR-132-3P, being involved in epilepsy and its emerging role in human cancer

SUPPLEMENTARY TABLES

Supplementary Table 1: Analysis of experimentally validated target gene of miR-212-3P/miR-132-3P; more detail can be found in sheet 2 and original data can be found in sheet 3.

See Supplementary File 1

Supplementary Table 2: The expression of all target geneS in human tiusse (DAVID).

See Supplementary File 2

Supplementary Table 3: Statistically enriched KEGG pathway(up) and biological processes categorization (below) of 951 validated genes of miR-132-3p and miR-212-3p.

See Supplementary File 3

Supplementary Table 4: The conservative genes predicted by targetscan and remaining genes of miR-132-3p and miR-212-3p (left) and the statistically enriched pathway and biological process categorization of remaining genes (right).

See Supplementary File 4

Supplementary Table 5: Excel shee1: Four group of genes for Venn diagrams (Figure 3c); Excel sheet2: string analysis for 14 intersection genes.

See Supplementary File 5

Supplementary Table 6: Excel sheet1 Differentially expressed genes of peirtumoral tissues between epilepsy and non-epilepsy. Excel sheet2: Comparison of genes between that analyzed by our and that coming from original article (the same gene with different probe sets was merged). Excel sheet3: the result of venn diagram in Figure 5c.

See Supplementary File 6

Supplementary Table 7: Execl sheet 1: Statistically enriched pathway against KEGG in 31.1% of DEGs; Execl sheet 2: statistically enriched GO-term (BP); Execl sheet 3: statistically enriched GO-term (CC); Execl sheet 4: statistically enriched GO-term (MF); Execl sheet 5: tissue expression of 177 DEGs; Execl sheet 6: CHROMOSOME of 177 DEGs.

See Supplementary File 7

Supplementary Table 8: Excel sheet 1: The representative example about the co-expression to support ceRNA hypothesis, positive co-expression of FRS2 and ceRNA SEMA6D was shown in Glioblastoma multiforme (GBM) and Colon and Rectal adenocarcinoma. Excel sheet 2: KEGG and String network consist of all the possible transcription factor compiled from Genecards and 68.9 % (393/570) DEGs.

See Supplementary File 8
